# Epidemiological trends and susceptibility patterns of bloodstream infections caused by *Enterococcus spp.* in six German university hospitals: a prospectively evaluated multicentre cohort study from 2016 to 2020 of the R-Net study group

**DOI:** 10.1007/s15010-024-02249-2

**Published:** 2024-04-30

**Authors:** Daniel Hornuss, Siri Göpel, Sarah V. Walker, David Tobys, Georg Häcker, Harald Seifert, Paul G. Higgins, Kyriaki Xanthopoulou, Beryl Primrose Gladstone, Chiara Cattaneo, Alexander Mischnik, Anna M. Rohde, Can Imirzalioglu, Janina Trauth, Moritz Fritzenwanker, Jane Falgenhauer, Petra Gastmeier, Michael Behnke, Evelyn Kramme, Nadja Käding, Jan Rupp, Silke Peter, Kristina Schmauder, Simone Eisenbeis, Winfried V. Kern, Evelina Tacconelli, Siegbert Rieg, DZIF R.-N. E. T. Study Group, L. A. Peña Diaz, G. Pilarski, N. Thoma, G. Peyerl-Hoffmann, H. Gölz, I. Joost, P. Mathé, M. Gillis, M. Vehreschild, J. Wille, B. Steffens, Y. Blum, M. Kleipass, D. Lenke, S. Herold, J. Schmiedel, A. Lengler, M. Buhl, F. Hölzl, A. Dinkelacker

**Affiliations:** 1 Division of Infectious Diseases, Department of Medicine II, Faculty of Medicine, Medical Center – University of Freiburg, University of Freiburg, 79106 Freiburg, Germany; 2 DZIF German Centre for Infection Research, Brunswick, Germany; 3 Division of Infectious Diseases, Department of Internal Medicine I, University Hospital Tübingen, Tübingen, Germany; 4 Institute for Medical Microbiology, Immunology and Hygiene, Faculty of Medicine and University Hospital Cologne, University of Cologne, Cologne, Germany; 5 Institute Für Clinical Microbiology and Hospital Hygiene, RKH Regionale Kliniken Holding Und Services GmbH, Ludwigsburg, Germany; 6 Institute for Medical Microbiology and Hygiene, University Medical Centre Freiburg, Freiburg, Germany; 7 Institute of Translational Research, CECAD Cluster of Excellence, University of Cologne, Cologne, Germany; 8 Department of Neonatology and Pediatric Intensive Care Medicine, University Medical Center Hamburg-Eppendorf, University Children’s Hospital, Hamburg, Germany; 9 Department of Infectious Diseases and Microbiology, University of Lübeck and University Hospital Schleswig-Holstein, Campus Lübeck, 23538 Lübeck, Germany; 10 Institute for Hygiene and Environmental Medicine, National Reference Centre for the Surveillance of Nosocomial Infections, Charité-University Hospital, Berlin, Germany; 11 Institute of Medical Microbiology, Justus Liebig University Giessen, Giessen, Germany; 12 Department of Internal Medicine (Infectious Diseases), Uniklinikum Giessen, Justus Liebig University Giessen, Giessen, Germany; 13 Institute of Medical Microbiology and Hygiene, University of Tübingen, Tübingen, Germany; 14 Division of Infectious Diseases, Department of Diagnostic and Public Health, University of Verona, Policlinico GB Rossi, Verona, Italy

**Keywords:** *Enterococcus*, VRE, Bloodstream infection, *E. faecium*, *E. faecalis*

## Abstract

**Purpose:**

To analyse recent epidemiological trends of bloodstream infections (BSI) caused by *Enterococcus* spp. In adult patients admitted to tertiary care centres in Germany.

**Methods:**

Epidemiological data from the multicentre R-NET study was analysed. Patients presenting with *E. faecium* or *E. faecalis* in blood cultures in six German tertiary care university hospitals between October 2016 and June 2020 were prospectively evaluated. In vancomycin-resistant enterococci (VRE), the presence of *vanA*/*vanB* was confirmed via molecular methods.

**Results:**

In the 4-year study period, 3001 patients with BSI due to *Enterococcus* spp*.* were identified. *E. faecium* was detected in 1830 patients (61%) and *E. faecalis* in 1229 patients (41%). Most BSI occurred in (sub-) specialties of internal medicine. The pooled incidence density of enterococcal BSI increased significantly (4.0–4.5 cases per 10,000 patient days), which was primarily driven by VRE BSI (0.5 to 1.0 cases per 10,000 patient days). In 2020, the proportion of VRE BSI was > 12% in all study sites (range, 12.8–32.2%). Molecular detection of resistance in 363 VRE isolates showed a predominance of the *vanB* gene (77.1%).

**Conclusion:**

This large multicentre study highlights an increase of BSI due to *E. faecium*, which was primarily driven by VRE. The high rates of hospital- and ICU-acquired VRE BSI point towards an important role of prior antibiotic exposure and invasive procedures as risk factors. Due to limited treatment options and high mortality rates of VRE BSI, the increasing incidence of VRE BSI is of major concern.

**Supplementary Information:**

The online version contains supplementary material available at 10.1007/s15010-024-02249-2.

## Introduction

*Enterococcus* species are commensals of the gastrointestinal system but may cause severe diseases including bloodstream infection (BSI) and endocarditis, which are predominantly caused by the species *Enterococcus faecalis* and *E. faecium*. Enterococci exhibit intrinsic resistances towards several antimicrobial classes including cephalosporins, lincosamides, fluoroquinolones, and aminoglycosides. Resistance to vancomycin is predominantly found in *E. faecium* but can also occur in *E. faecalis*. Treatment options are limited in both, vancomycin-susceptible *E. faecium* (VSEfm) and vancomycin-resistant *E. faecium* (VREfm) [1].

According to the Global Burden of Disease Study, *E. faecium* and *E. faecalis* range within the top 10 pathogens associated with global mortality of infectious syndromes in 2019 [2]. In particular, an estimated 100,000 to 250,000 global deaths are likely to be associated with antimicrobial resistance of *E. faecium* [3]. Compared to the quite stable incidence of infections due to carbapenem-resistant Gram-negative bacteria, and declining incidence of invasive infections due to methicillin-resistant *Staphylococcus aureus* (MRSA), epidemiological data from Germany have shown an increase of invasive infections caused by vancomycin resistant enterococci (VRE), particularly in intensive care units (ICU) since 2007 [4–7]. Mortality of severe BSI or infective endocarditis due to enterococci may range between 20 and 30% and persistent bacteraemia was shown to be a risk factor for poor outcomes in VRE BSI [8–10]. A recent meta-analysis of data from Europe showed a pooled incidence of nosocomial enterococcal infections up to 24.8 cases per 1000 patients over 10 years (2010–2020), which was associated with high mortality rates of 21.9% for all enterococci and 33.5% for VRE, respectively [10].

Invasive infections due to enterococci may be accelerated by prior exposure to antimicrobial agents leading to an increased density of gastrointestinal and mucocutaneous colonization with *Enterococcus* spp. Subsequently foreign devices like intravascular catheters pose a risk of hospital-acquired infections [1, 11]. The prevalence of VRE colonization of patients is increasing in the US and in Europe, which, in Germany, is paralleled by expansion of the sequence type (ST) 117 [12–17]. Yet, the potential consequences with regard to the occurrence of BSI are not known.

Within this study we aimed to investigate current epidemiological trends and resistance profiles in BSI due to *Enterococcus* spp. in patients at six German university hospitals with a focus on critically ill, often comorbid patients that represent a major subgroup at risk for enterococcal infections over a period of 4 years.

## Methods

### Study participants, settings, and definitions

The study was conducted at six German tertiary care university hospitals (centre 1–6) as a multicentre prospective non-interventional cohort study (R-Net) performed within the German Center for Infectious Diseases Research (DZIF). Patients with blood cultures growing at least one target pathogen (comprising *Acinetobacter baumannii*, *Enterobacter* spp., *Escherichia coli*, *Klebsiella* spp., *Pseudomonas aeruginosa*, *Staphylococcus aureus*, *E. faecium* and *E. faecalis*) aged ≥ 18 years and admitted to hospital between October 2016 and June 2020 were included. Patients hospitalized in departments of dermatology, ophthalmology and psychiatry/psychosomatics were excluded. Only data of patients with positive blood cultures for *E. faecium* or *E. faecalis* were analysed in this study. Age, date of admission, and the blood culture sampling department were recorded.

Polymicrobial bloodstream infection was defined as detection of ≥ 2 different R-Net target pathogens in one blood culture set. Repeated detection of identical enterococcal isolates in blood cultures obtained within ≤ 30 days was defined as one episode, while detection > 30 days was considered as a new episode (except cases of ongoing persistent bacteraemia). Hospital-acquired BSI was defined as detection of a target pathogen in blood cultures taken ≥ 48 h after admission. Data on admission (patient cases) and patient days (occupied bed days) were obtained from each hospital for the study period from October 2016 to December 2019.

### Bacterial isolate collection and antimicrobial susceptibility testing

Aerobe and anaerobe blood cultures were collected at the discretion of the clinicians using the locally available blood culture systems (e.g., BD BACTEC™, Becton Dickinson, Heidelberg, Germany, or BacT/ALERT®, bioMérieux, Nürtingen, Germany). Incubation of blood cultures and identification of pathogens were performed in local microbiology laboratories according to standard protocols. In vitro susceptibility testing of ampicillin, linezolid, teicoplanin and vancomycin was conducted using standardized testing procedures (e.g. VITEK2 AST P592 cards and Etest, bioMérieux). Minimum inhibitory concentrations (MICs) were interpreted using EUCAST breakpoints for *Enterococcus* spp. (Version 10.0, January 2020). Vancomycin resistance was defined as MIC > 4 mg/l. All clinical isolates obtained within the study period were included for phenotypic susceptibility testing. In a subset of VRE isolates, *vanA*/*vanB* was confirmed by PCR.

### Statistical methods

For statistical analysis of differences between two groups Mann–Whitney-*U*-Test and for differences between multiple groups Kruskal–Wallis-Test or one-way-ANOVA were performed. Trends were shown using simple linear regression models. Logistic regression models were used for identifying independent risk factors. Statistical significance was defined as *p* < 0.05. For statistical analyses and graphic illustrations GraphPad Prism Version 9.3.1 (GraphPad Software, San Diego, USA) was used.

### Ethics

The R-Net study was approved by the respective institutional review boards at each study site (approval number 16-309).

## Results

**Fig. 1:**
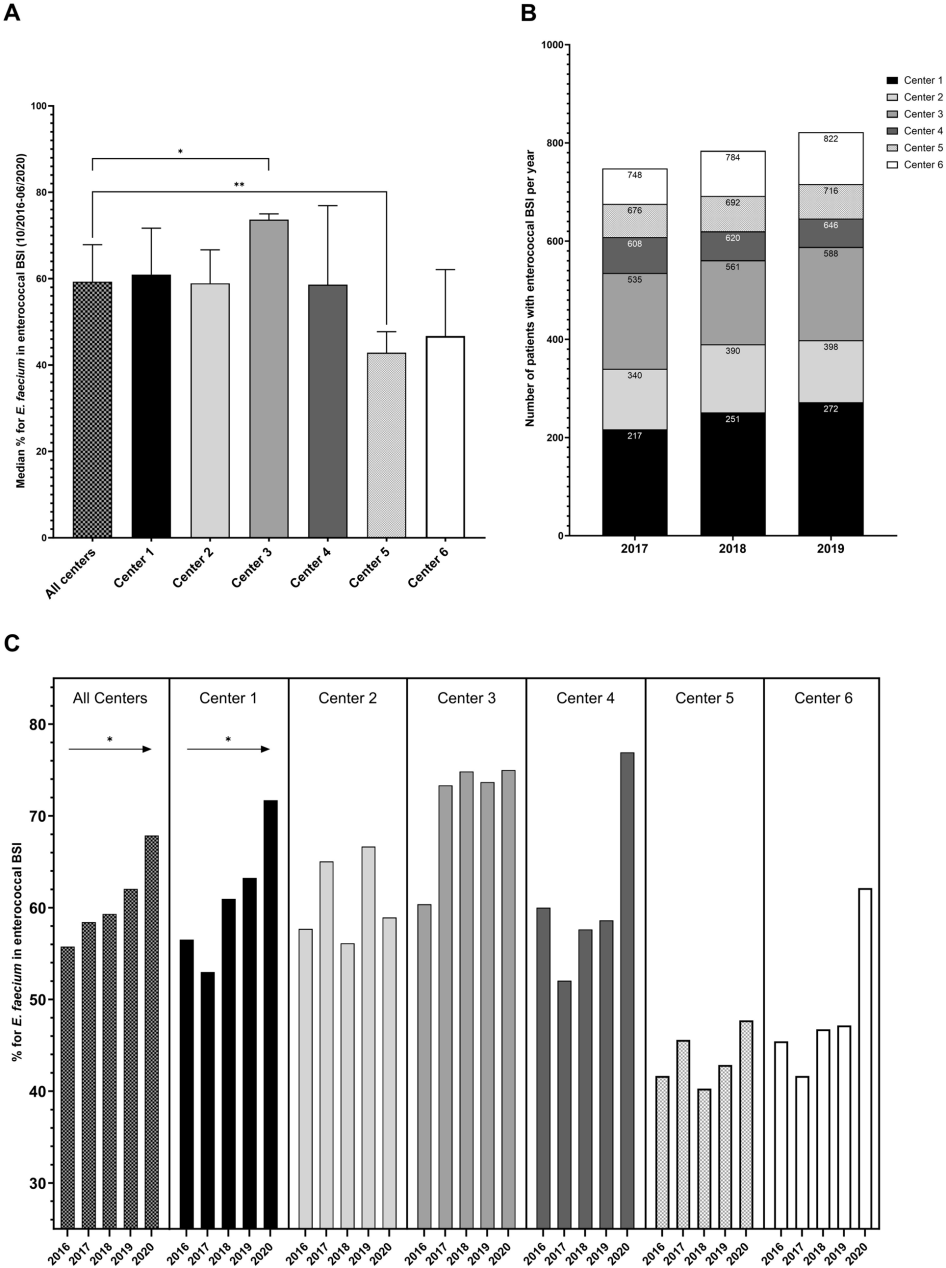
Distribution of enterococcal species and temporal trends per study site. **A** Overall median proportion of *E. faecium* in enterococcal BSI over the whole study period per study site (with 95 CI of median; Mann-Whitney-U-Test p ≤ 0.05 [*], *p* ≤ 0.01 [**]). **B **Total annual number of patients with enterococcal BSI in the participating study centres. **C** Temporal trends in the pooled proportion of *E. faecium* in enterococcal BSI over time (Linear regression *p* ≤ 0.05 [*])

A total of 3001 patients with enterococcal BSI were identified. In 72 patients a persistent enterococcal BSI was detected (70 patients with two and two patients with three positive blood cultures, but no new BSI episode), resulting in a total of 3075 clinical blood culture isolates (Table 1). Of note, 58 patients had BSI with both *E. faecalis* and *E. faecium.* Polymicrobial BSI (with other R-Net target pathogens) occurred in about 16% of patients with higher rates in *E. faecalis* BSI (24.4%) than in BSI due to *E. faecium* (13.1%; *p* = 0.016). Overall, *E. faecium* was the predominant species (1830 cases [61.0%] compared to 1229 cases with *E. faecalis* [41.0%]), but the proportions varied substantially between study sites (range, 43.6–71.5%; *p* < 0.0001) (Fig. 1A). VRE BSI were detected in a total of 569 patients (19.0%), ranging from 8.6% to 22.2% between centres (Table 1). The vast majority of VRE BSI was caused by *E. faecium*, with only three VRE BSI caused by *E. faecalis*.

**Table 1:** Demographics and characteristics of patients with *Enterococcus* spp. BSI

Characteristics	*Enterococcus* spp*.*	*E. faecalis*	*E. faecium*	VRE
No. of patients with BSI	3001	1229^2^ (41.0%)	1830^2^ (61.0%)	569^3^ (19.0%)
All clinical isolates^1^	3075	1230 (40.0%)	1845 (60.0%)	570 (18.5%)
Cases/1000 admissions (median)	1.97 (IQR 1.88–2.15)	0.87 (IQR 0.82–0.88)	1.15 (IQR 1.07–1.32)	0.32 (IQR 0.24–0.47)
Cases/10,000 patient-days (median)	4.15 (IQR 4.0–4.45)	1.81 (IQR 1.76–1.84)	2.44 (IQR 2.25–2.73)	0.68 (IQR 0.51–0.97)
Age (years, median)	67 (IQR 57–77)	71 (IQR 61–79)	65 (IQR 56–74)	65 (IQR 55–73)
Male sex, n (%)	2000 (66.6%)	848 (69.0%)	1190 (65.0%)	367 (64.5%)
No. of patients with polymicrobial BSI (≥ 2 pathogens)	481 (16.0%)	300 (24.4%/62.4%)	239 (13.1%/49.7%)	72 (12.7%/15.0%)
Department/Unit				
Internal medicine	1888 (62.9%)	693 (56.4%/36.7%)	1230 (67.2%/65.2%)	397 (69.8%/21.0%)
Haematology/oncology	316 (10.5%)	69 (5.6%/21.8%)	250 (13.7%/79.1%)	74 (13.0%/23.4%)
Surgery	757 (25.2%)	339 (27.6%/44.8%)	438 (23.9%/57.9%)	132 (23.2%/17.4%)
Visceral surgery	444 (14.8%)	180 (14.7%/40.5%)	277 (15.1%/62.4%)	73 (12.8%/16.4%)
Neurology, neurosurgery	105 (3.5%)	74 (6.0%/70.5%)	33 (1.8%/31.4%)	9 (1.6%/8.6%)
Other	251 (8.4%)	123 (10.0%/49.0%)	129 (7.1%/51.4%)	31 (5.4%/12.4%)
Level of care				
ICU	1366 (45.5%)	428 (34.8%/31.3%)	962 (52.6%/70.4%)	331 (58.2%/24.2%)
General ward	1623 (54.1%)	793 (64.5%/48.9%)	864 (47.2%/53.2%)	237 (41.7%/14.6%)
n.a.^4^	12 (0.4%)	8 (0.7%/66.7%)	4 (0.2%/33.3%)	1 (0.2%/8.3%)
Acquisition of BSI				
Hospital-acquired	2125 (70.8%)	622 (50.6%/29.3%)	1540 (84.2%/72.5%)	491 (86.3%/23.1%)
ICU	1103 (36.8%)	251 (20.4%/22.8%)	870 (47.5%/78.9%)	302 (53.1%/27.4%)
Regular ward	1022(34.1%)	371 (30.2%/36.3%)	670 (36.6%/65.6%)	189 (33.2%/18.5%)

^1^Overall number of clinical isolates detected in blood cultures. 72 patients showed multiple detection of the same enterococcal BSI

^2^58 patients had BSI with both *E. faecium* and *E. faecalis*

^3^One patient had two positive blood cultures with VRE

^4^n.a.: not available

The median age of patients with enterococcal BSI was 67 years (Table 1). Patients with *E. faecalis* BSI had a higher median age than patients with *E. faecium* BSI (71 vs. 65 years, *p* < 0.0001) and were slightly more often of male sex (69% vs. 65%, *p* = 0.008).

Most enterococcal BSI were hospital-acquired (70.8%), observed in internal medicine departments (62.9%) and among patients admitted to general wards (54.1%) (Table 1), with similar rates at all sites (Supplemental Fig. 1). Hospital acquisition was most frequent for *E. faecium* (84.2%), in particular for VRE (86.3%). The highest proportions of *E. faecium* cases were seen in departments of haematology-oncology (79.1%, range between centres, 71.4% to 83.3%). Interestingly, the lowest proportion of *E. faecium* (31.4%) was seen in neurology and neurosurgery units.

The median overall incidence was 1.97 enterococcal BSI per 1000 admissions (range 1.87–2.18) corresponding to an incidence density of 4.15 BSI episodes per 10,000 patient-days (range 3.99–4.51) during the study period (Table 1). The incidence of *E. faecium* BSI was significantly higher with 1.15 cases (vs. 0.87 cases in *E. faecalis* BSI) per 1000 patients (*p* = 0.029) while the incidence density was 2.44 *E. faecium* BSI episodes (vs. 1.81 *E. faecalis* BSI episodes) per 10,000 patient-days (*p* = 0.029). Yet, detection of *E. faecium* and VRE was particularly pronounced in ICU patients (9.15 and 3.27 BSI episodes per 10,000 patient-days, respectively).

### Temporal trends in distribution of *Enterococcus* spp. BSI isolates

**Fig. 2:**
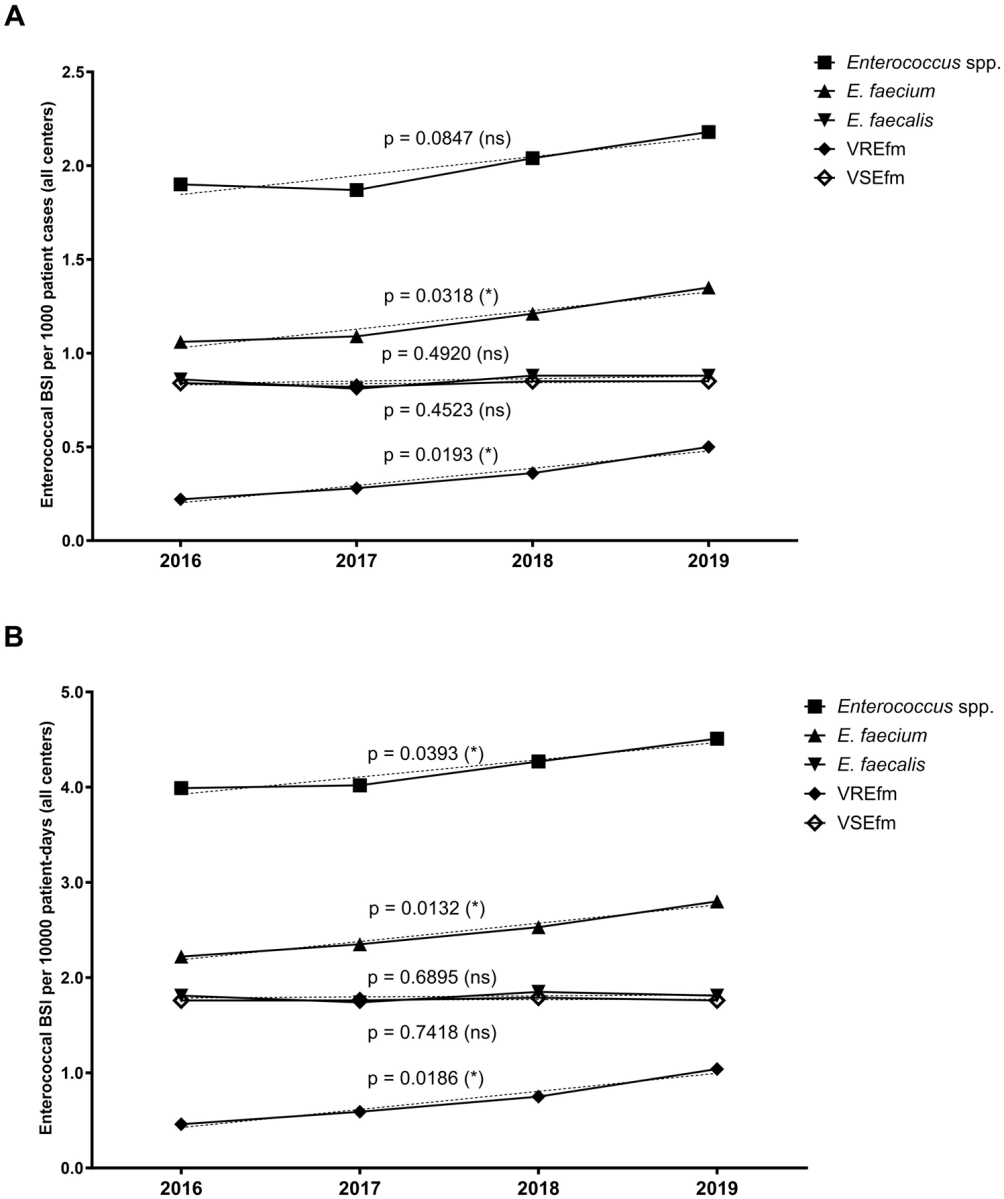
Incidence and incidence density of BSI due to *Enterococcus* spp., *E. faecium*, *E. faecalis*, vancomycin-susceptible *E. faecium* (VSEfm) and vancomycin-resistant *E. faecium* (VREfm); statistical significance calculated with linear regression (*p* ≤ 0.05 [*], *p* ≤ 0.01 [**]). **A** Annual trends for pooled incidence of enterococcal BSI (no. of BSI per 1000 admissions). **B** Annual trends for pooled incidence density of enterococcal BSI (no. of BSI per 10,000 patient-days)

**Fig. 3:**
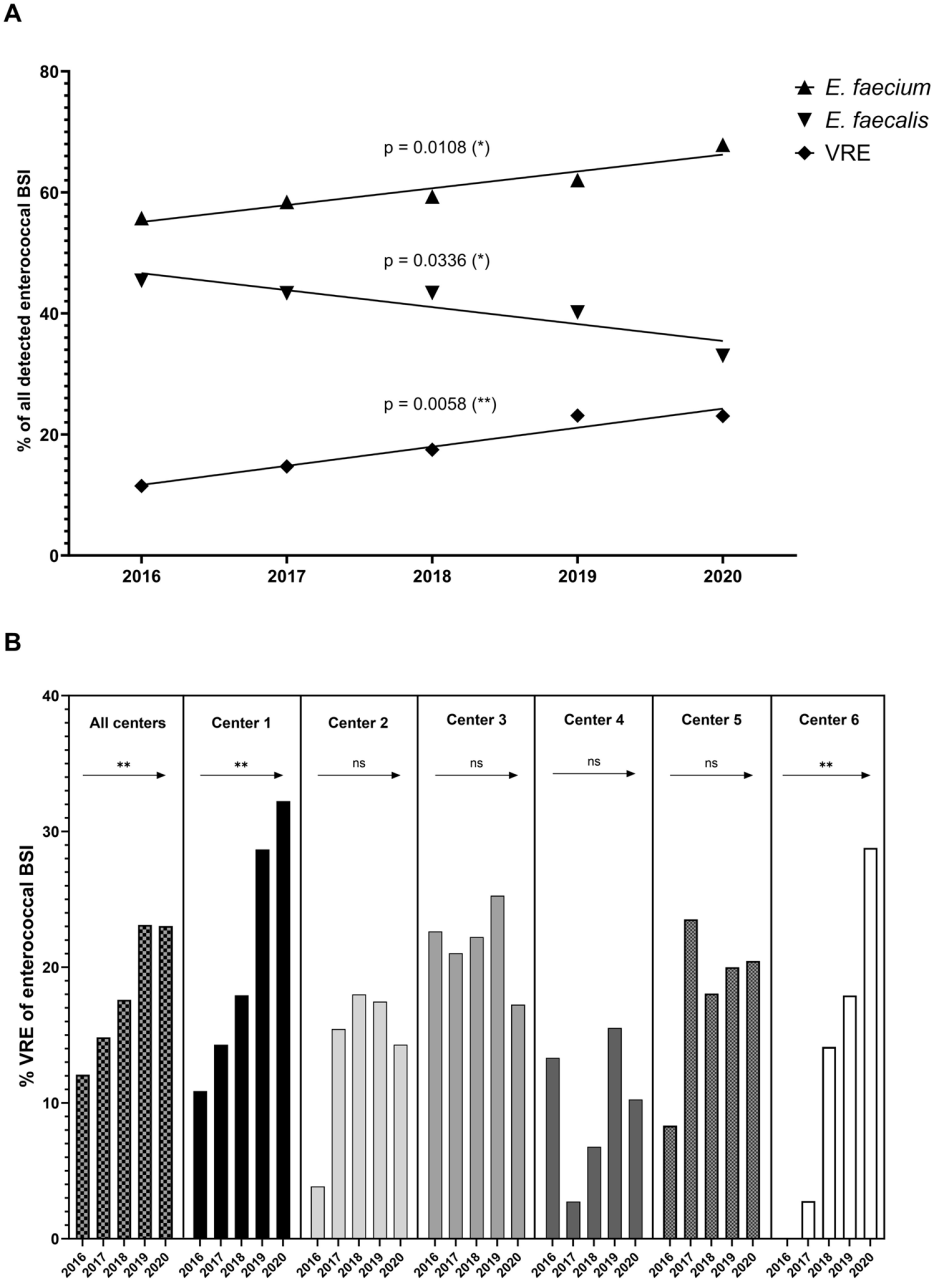
Overall and local trends of VRE BSI based on pooled proportional analyses; statistical significance calculated with linear regression (*p* ≤ 0.05 [*], *p* ≤ 0.01 [**]). **A** Annual trends for bloodstream infections due to *E. faecium*, *E. faecalis* and VRE. **B** Local annual trends for bloodstream infections due to VRE per study centre

The aggregated number of enterococcal BSI in all centres (data from 2017 to 2019) showed an overall increase of 4.8% each year with 748 patients in 2017, 784 patients in 2018 and 822 patients in 2019 (Fig. 1B), but with divergent trends for *E. faecium* and *E. faecalis*. The proportion of BSI due to *E. faecium* increased significantly from 55.8% in 2016 to 67.9% in 2020 (*p* = 0.0108), however with differing trends in the study sites (Fig. 1C). The overall incidence and incidence density of enterococcal BSI increased, which was driven by increasing numbers of *E. faecium* and VREfm BSI (Figs. 2 and 3). Conversely, both the incidence and incidence density of VSEfm and *E. faecalis* BSI did not show any significant trends during the observation period and the proportions of *E. faecalis* BSI decreased (from 45.4% to 33.0%) while those of *E. faecium* BSI increased (driven by the increasing proportion of VREfm BSI from 12.1% in 2016 to 23.0% in 2020) (Fig. 2 and 3B). Linear regression analysis showed no increase of patients’ median age during the observation time, but logistic regression analysis showed a lower age as independent risk factor for VRE bloodstream infection (*p* < 0.001). In addition, an increase of numbers of enterococcal BSI was observed especially in departments of internal medicine in some study centres (Supplemental Fig. 1). The percentage rate of VREfm BSI (compared to all enterococcal BSI) increased until 2019 to more than 25% in all departments of haematology-oncology at the various study sites (no data for centre 5 available).

### In vitro antibiotic susceptibility and molecular resistance

A total of 3075 enterococcal isolates from 3001 patients underwent phenotypic susceptibility testing. 1181 of 1189 *E. faecalis* isolates (99.3%), which were tested for ampicillin susceptibility, were susceptible or intermediate susceptible (Table 2) (41 *E. faecalis* isolated were not tested). Vancomycin resistance was detected in only three *E. faecalis* isolates (0.3%). In contrast, 1705 of 1811 (94.1%) *E. faecium* isolates were resistant to ampicillin. Among *E. faecium* isolates, vancomycin susceptibility was found in 68.5%, and teicoplanin susceptibility in 87.6% of isolates. Of 498 tested VRE isolates, 307 (61.4%) tested susceptible to teicoplanin. An additional PCR-based analysis of the resistance genes *vanA* and *vanB* was performed in a subgroup of 363 VRE isolates, (Table 3). *VanA* was detected in 81 isolates (22.3%) and *vanB* in 280 isolates (77.1%), while two isolates had both *vanA* and *vanB.* Teicoplanin MIC testing of 302 PCR-tested isolates showed an overall susceptibility of 220 isolates (72.8%). Isolates with *vanA* were predominantly resistant to teicoplanin (76/78 isolates, 97.4%) while 218 of 222 *vanB*-positive isolates (98.2%) were susceptible to teicoplanin. Low rates of linezolid resistance were found in both *E. faecalis* (10/1187, 0.8%) and *E. faecium* (38/1803, 2.1%), including VRE (18/566, 3.2%) without significant changes during the study period (Supplemental Fig. 2).

**Table 2:** Susceptibility of *E. faecium*, *E. faecalis* and VRE bloodstream infection isolates

	No. of isolates tested		
Agent	*E. faecalis*	*E. faecium*	VRE
All clinical isolates	1230	1845	570
Ampicillin testing	1189	1811	566
S/I	1181 (99.3%)	106 (5.9%)	2 (0.4%)
R	8 (0.7%)	1705 (94.1%)	564 (99.6%)
Vancomycin testing	1193	1800	570
S	1190 (99.7%)	1233 (68.5%)	0 (0%)
R	3 (0.3%)	567 (31.5%)	570 (100.0%)
Teicoplanin testing	959	1563	498
S	958 (99.9%)	1368 (87.5%)	307 (61.4%)
R	1 (0.1%)	195 (12.5%)	191 (38.4%)
Linezolid testing	1187	1803	566
S	1177 (99.2%)	1765 (97.9%)	548 (96.8%)
R	10 (0.8%)	38 (2.1%)	18 (3.2%)

**Table 3:** Molecular detection of *vanA* and *vanB* and susceptibility patterns of 363 VRE isolates

Agent	All VRE isolates	*vanA*	*vanB*	*vanA/B*
No. of isolates	363	81 (22.3%)	280 (77.1%)	2 (0.6%)
Teicoplanin susceptibility (no. of isolates tested)	302	78	222	2
S	220 (72.8%)	2 (2.6%)	218 (98.2%)	0
R	82 (27.2%)	76 (97.4%)	4 (1.8%)	2 (100%)

## Discussion

Our analysis highlights important aspects concerning epidemiological features of enterococcal BSI in a tertiary care setting in Germany: (i), we observed an overall increasing trend of BSI due to *E. faecium*, which is primarily driven by VREfm; (ii), VRE BSIs are predominantly acquired in the hospital, and in particular in the ICU and are most frequent in internal medicine departments; (iii), number, proportion and incidence of VRE BSI as well as temporal trends show a significant variability among the different study centres; and (iv), the epidemiology of VRE in Germany is dominated by a high prevalence of the *vanB* gene.

Increasing rates of vancomycin resistance in enterococci has been reported in Germany in several recent studies [4–6] most of which investigated a variety of hospital-acquired infections comprising BSI, urinary tract, and surgical site infections. Interestingly, an analysis of VRE colonization in hospitalized patients of the same study centres revealed an increasing admission prevalence from 0.8% to 2.6% between 2014 and 2018 [16]. Our findings are in concordance with previous analyses and suggest a correlation between increasing colonization of hospitalized patients and occurrence of VRE BSI [4–6, 15].

The present study clearly shows that *E. faecium* has replaced *E. faecalis* as leading cause of BSI in tertiary care centres. Compared to the annual ECDC reports on invasive VRE isolates in European countries, our data show a higher proportion of VRE BSI. A decline of VRE BSI as described in the ECDC report of 2020 was not evident from our data of German tertiary care hospitals [12]. Moreover, in comparison to Brinkwirth et al., who also analysed epidemiological data of hospital-acquired enterococcal infections in Europe, we observed a higher incidence and a higher incidence-density for hospital-acquired enterococcal BSI and VRE BSI, which may be explained with our focus on German tertiary care centres but also with differences in prevalence of VRE colonization between different countries [10, 18]. However, we found a significant variation in the proportion of BSIs due to *E. faecalis*, VSE and VRE among different study centres, which is also present in the ECDC reports and other studies [4, 10, 12]. Although all recruiting centres were tertiary care hospitals, differences concerning the number of haematology/oncology patients or ICU patients may explain these variations. Despite local differences, an overall trend towards a predominance of *E. faecium* in enterococcal BSI (primarily driven by the increase of VRE BSI) was evident, which is in contrast to an earlier study from 2012 indicating *E. faecalis* as causative pathogen in 60–95% of enterococcal BSI in German or European hospitals [19–21]. Beside analysing data from tertiary care hospitals, the overall increase of *E. faecium* may also be driven by the rapid expansion of two VRE STs, i.e., ST80 and ST117, first detected in Germany in 2015 [17]. The expansion especially of sequence type ST117 may also be associated with the predominance of the *vanB* gene, which was detected in about 77% of all VRE isolates in our analysis. The resulting susceptibility rate of > 72% renders teicoplanin a potentially valid treatment option of VRE BSI in Germany, although the most widely used agents in a recent study at tertiary care centres were daptomycin and linezolid (of which resistance rates remained unchanged at ~ 3% in the current study) [22]. However, conclusions based on these data may be limited due to incomplete teicoplanin susceptibility data (87% of VRE isolates) and molecular analysis of *vanA* and *vanB* only in a subset of isolates.

Strengths of the current study include the prospective study design, the stratification by department and the high number of cases, which allows to highlight distinct epidemiological aspects of enterococcal BSI in diverse centres/regions and in ICU und non-ICU settings over the study period. The multicentre analysis over several years also allows for differentiation between general trends and centre effects, thereby minimizing the influence of local outbreaks. Due to the lack of data, our study provides no information on clinical outcomes of the observed enterococcal BSI, representing a limitation in assessing the overall burden. Further limitations are incomplete data for the years 2016 and 2020 which may lead to uncertainties of measurements in these years. In addition, our analysis focuses on tertiary care hospitals, which may overestimate the ratio of *E. faecium* and VRE BSI compared to hospitals of primary or secondary care levels and limits generalizability of our data.

The increasing incidence of VRE BSI and the associated burden of disease is contrasted by a scarcity in well-designed clinical studies on optimized management of enterococcal and VRE BSI. Recent analyses indicate treatment success rates of VRE BSI varying between 50 and 80% and mortality rates of 20 to 30%. Furthermore, the risk of death is considered ~ 2.7 fold higher in patients with VRE-bacteraemia compared to VSE-bacteraemia [22–26]. The high rates of hospital-acquired infections and allocation of *E. faecium* and VRE BSI to distinct clinical disciplines underline the impact of comorbidities and interventional procedures on the occurrence of these invasive infections. Besides further clinical studies to evaluate optimized therapeutic approaches and management bundles to improve clinical outcomes of VRE BSI, an intensified effort on prevention of invasive VRE infections also has to be addressed in clinical practice. Valuable tools for prevention might be expansion of hygiene measures in patients undergoing complex procedures to minimize potential portals of entry but also reducing unnecessary antibiotic prescriptions by establishing or expanding local antimicrobial stewardship programs.

## Conclusion

These findings are noteworthy and shed light on a difficult-to-treat infection and challenging task in the years to come. While resistance rates of MRSA or multidrug-resistant Enterobacterales seem to decline or remain stable, enterococcal BSI and particularly VRE BSI are on the rise. VRE-BSI were predominantly acquired in hospital, particularly in departments of internal medicine or on ICUs, pointing to an important role of prior antibiotic exposure and invasive procedures as risk factors. Due to limited treatment options and high mortality rates of VRE BSI, the increasing incidence of VRE BSI is of major concern and necessitates further efforts in clinical studies and measures for prevention of invasive VRE infections.

## Supplementary Information

Below is the link to the electronic supplementary material.

**Supplementary file 1:** (JPG 1509 KB)

**Supplementary file 2:** (JPG 523 KB)

## Data Availability

No datasets were generated or analysed during the current study.
