# Supplementary figures and images for: Epidemiological trends and susceptibility patterns of bloodstream infections caused by Enterococcus spp. in six German university hospitals: a prospectively evaluated multicentre cohort study from 2016 to 2020 of the R-Net study group
Source: Infection. 2024 Apr 30;52(5):1995–2004. doi: 10.1007/s15010-024-02249-2 (PMC11499396; doi:10.1007/s15010-024-02249-2)

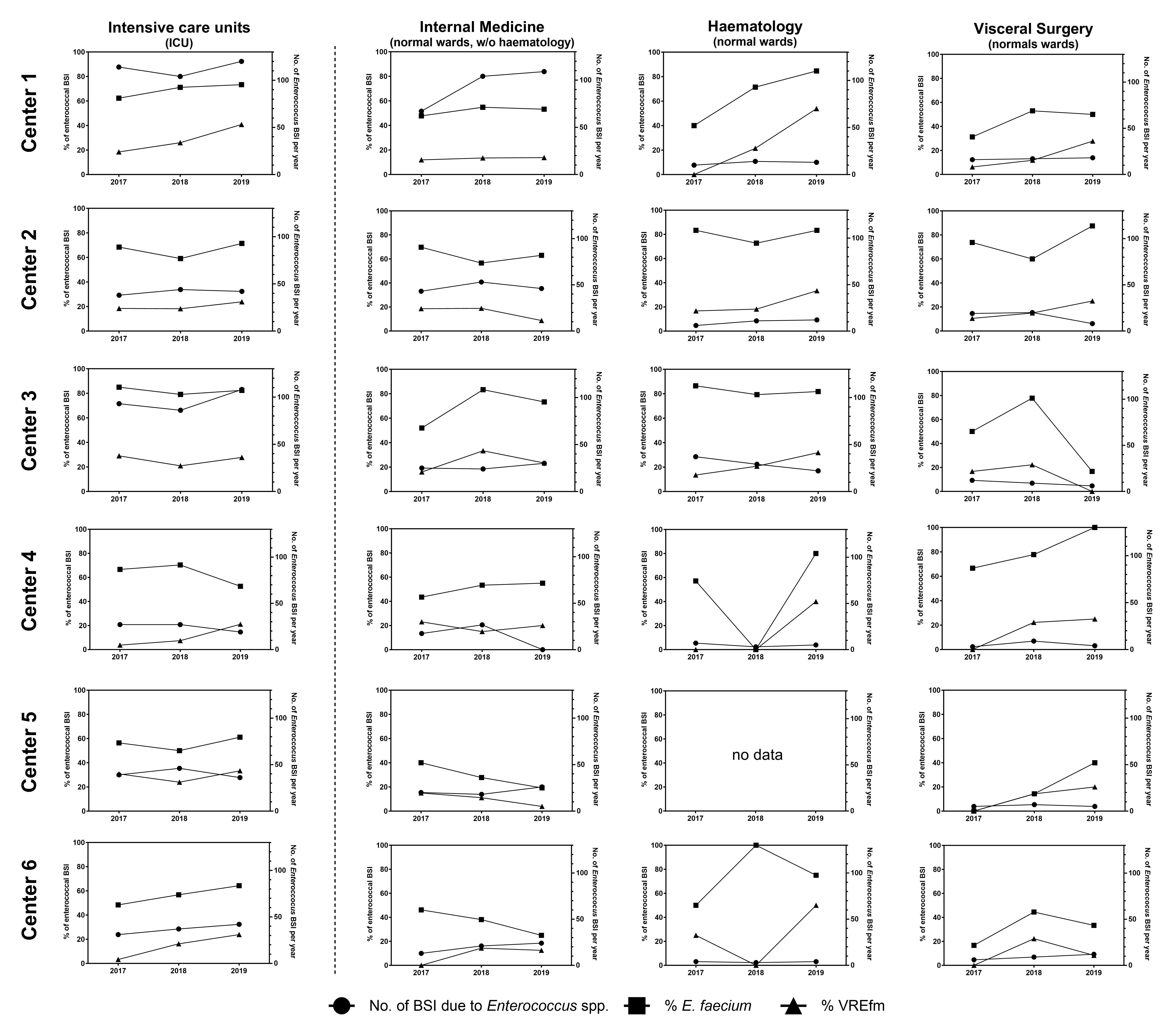

Supplement: Supplementary file 1 — (JPG 1509 KB) [file 15010_2024_2249_MOESM1_ESM.jpg]

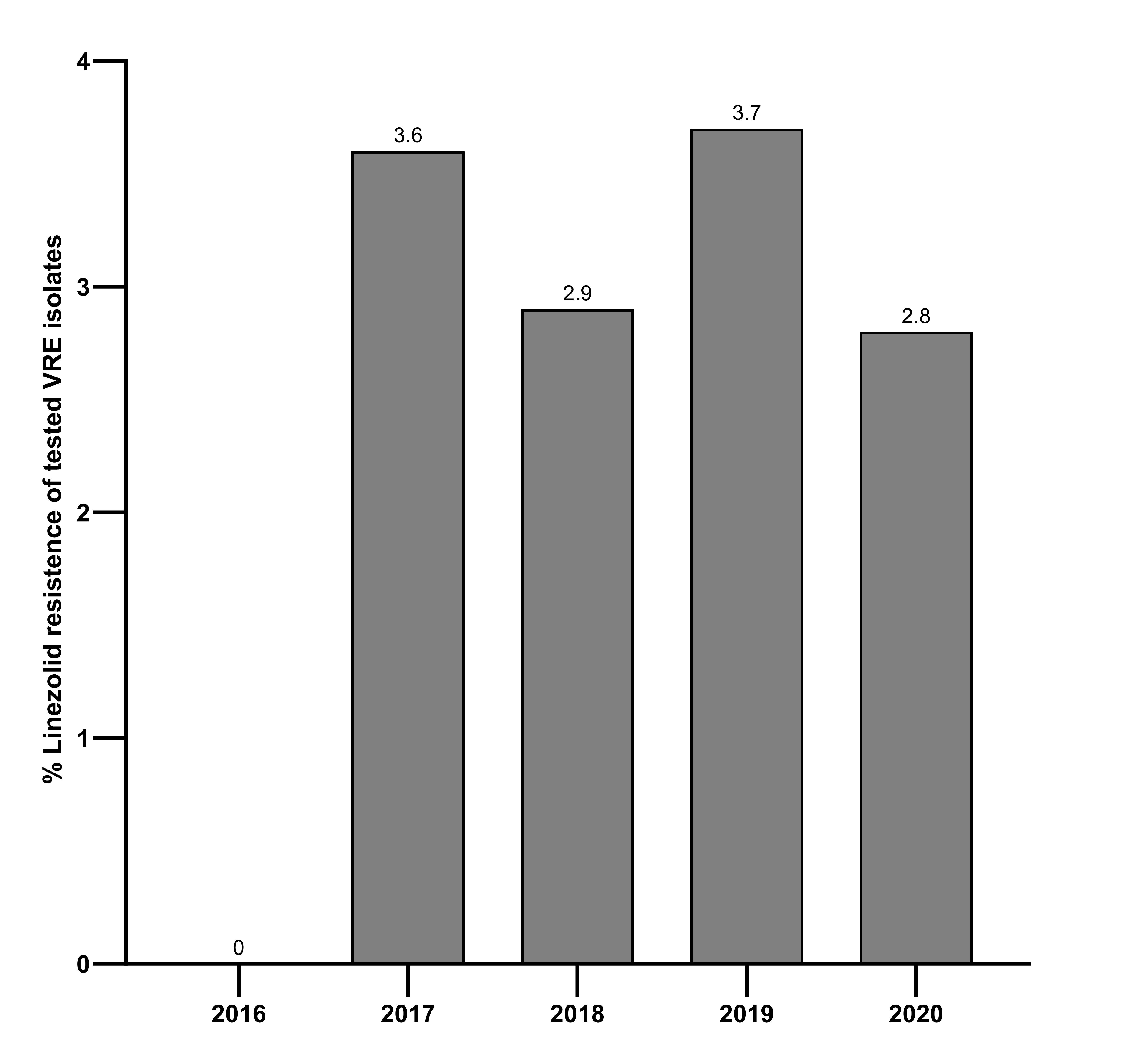

Supplement: Supplementary file 2 — (JPG 523 KB) [file 15010_2024_2249_MOESM2_ESM.jpg]
